# Decellularized dental pulp extracellular matrix for pulp-dentin regeneration: a narrative review of processing, bioactivity, delivery, preclinical evidence, and translational challenges

**DOI:** 10.3389/fdmed.2026.1962311

**Published:** 2026-09-25

**Authors:** Xinli Zhang, Mengjie Dong

**Affiliations:** Hangzhou Stomatology Hospital, Hangzhou, Zhejiang, China

**Keywords:** decellularized dental pulp extracellular matrix, DPECM, extracellular matrix hydrogel, pulp-dentin complex, regenerative endodontics

## Abstract

Regenerative endodontics is intended to bring living function back to the pulp-dentin complex. That means restoring blood supply, neural support, immune activity, and dentin formation, not simply filling the canal with repair tissue. Blood-clot-based procedures can promote healing, but the tissue that forms is often uneven and does not closely resemble normal pulp. This limitation has drawn attention to decellularized dental pulp extracellular matrix (DPECM). DPECM keeps part of the native three-dimensional framework together with collagens, glycosaminoglycans, matricellular proteins, and matrix-bound signals. The objective of this narrative review is to critically evaluate how native dental pulp-derived DPECM is prepared and delivered, what biological effects and candidate mechanisms are directly supported, and what evidence gaps limit clinical translation. A focused PubMed search from database inception to 31 July 2026 used terms for dental pulp, decellularized extracellular matrix, DPECM, and regenerative endodontics; reference-list screening supplemented the search, and 101 articles were retained for narrative synthesis. Across the available, predominantly preclinical literature, DPECM has generally been associated with dental pulp stem-cell adhesion and migration, several differentiation programs, and angiogenic, neural-marker, and immune-remodeling outcomes. Whole-pulp scaffolds preserve tissue architecture more faithfully. Solubilized hydrogels, microgels, and photocrosslinked composites are easier to place in irregular root canals, although some structural information is lost during processing. Comparative omics has started to explain why pulp-derived matrix may behave differently from other matrices. Candidate tenascin-C/Notch signaling is one example. Another recurring theme is the coupling of macrophage behavior with vascular and odontogenic repair. Recent orthotopic canine studies provide stronger preclinical evidence of pulp-like tissue with dentinogenic, vascular-marker, and neural-marker outcomes, but not restored function. Even so, most evidence still comes from cell culture, ectopic implantation, or short animal experiments. Differences in tissue source, processing, controls, and outcome definitions make effect sizes difficult to compare. The next stage of the field will require agreed quality attributes, practical potency assays, infected-root models, long-term functional outcomes, and direct comparison with current regenerative procedures. DPECM remains a predominantly preclinical strategy: no direct human clinical evaluation of a DPECM-based therapeutic product was identified, and translation will require reproducible manufacturing, disease-relevant testing, and durable functional outcomes.

## 1 Introduction

Dental pulp is a vascular and richly innervated connective tissue. It forms dentin, contributes to sensation and defense, supports immune surveillance, and helps maintain tooth homeostasis. Conventional root canal treatment controls infection and often preserves the tooth. Still, it removes the living pulp and replaces it with an inert filling, so the original biological functions are not restored. Regenerative endodontic procedures (REPs) take a different approach. They try to establish living tissue inside the canal, especially in immature permanent teeth whose roots stopped developing after pulp necrosis. Clinical reports describe apical healing and continued root maturation, but histology varies considerably. For this reason, revitalization, repair, and true regeneration should not be treated as the same outcome (1).

Tissue engineering usually brings together responsive cells, signaling cues, and a scaffold. The scaffold does much more than occupy space. It affects cell attachment, movement of oxygen and nutrients, degradation, release of bound factors, and the mechanical signals sensed by recruited or transplanted cells (2). Cell-free approaches avoid *ex vivo* cell expansion by drawing progenitor cells from the host. Their success, however, depends heavily on whether the matrix can guide cell homing, survival, and lineage decisions (3). Decellularized extracellular matrix (dECM) is made by removing cells and nuclear material while trying to retain the tissue's structure and biochemical information. Physical, chemical, and enzymatic treatments can clear cellular material, but none is neutral. Collagen, glycosaminoglycans, basement-membrane proteins, and matrix-bound growth factors may all be altered during processing (4). Breakdown products from dECM can also influence constructive remodeling, angiogenesis, and immune responses. This behavior separates dECM from purified collagen and from biologically inert polymers (5). The familiar benchmark less than 50 ng of double-stranded DNA per milligram of dry ECM, DNA fragments shorter than 200 base pairs, and no visible nuclei are useful screening criteria. They do not, on their own, prove sterility, safety, or biological potency (6).

Pulp-derived ECM is appealing because it may preserve signals suited to odontoblast differentiation, vascular growth, and neural support. Yet DPECM is not one uniform product. An intact pulp scaffold is biologically and physically different from a milled porous matrix, a pepsin-digested hydrogel, a microsphere preparation, or a polymer-reinforced composite. These formats differ in architecture, mechanics, degradation, and how cells encounter matrix cues. We first examine how DPECM is prepared and how cells respond to it. We then consider delivery systems, experimental evidence, proposed signaling mechanisms, clinical context, and the work still needed. Accordingly, the guiding question is: to what extent do current data directly support the processing, biological activity, candidate mechanisms, delivery performance, and translational readiness of native tissue-derived DPECM, as distinct from related cell-derived ECM and other regenerative approaches? An overview of DPECM preparation, delivery, and regenerative mechanisms is summarized in Figure 1.

**Figure 1:**
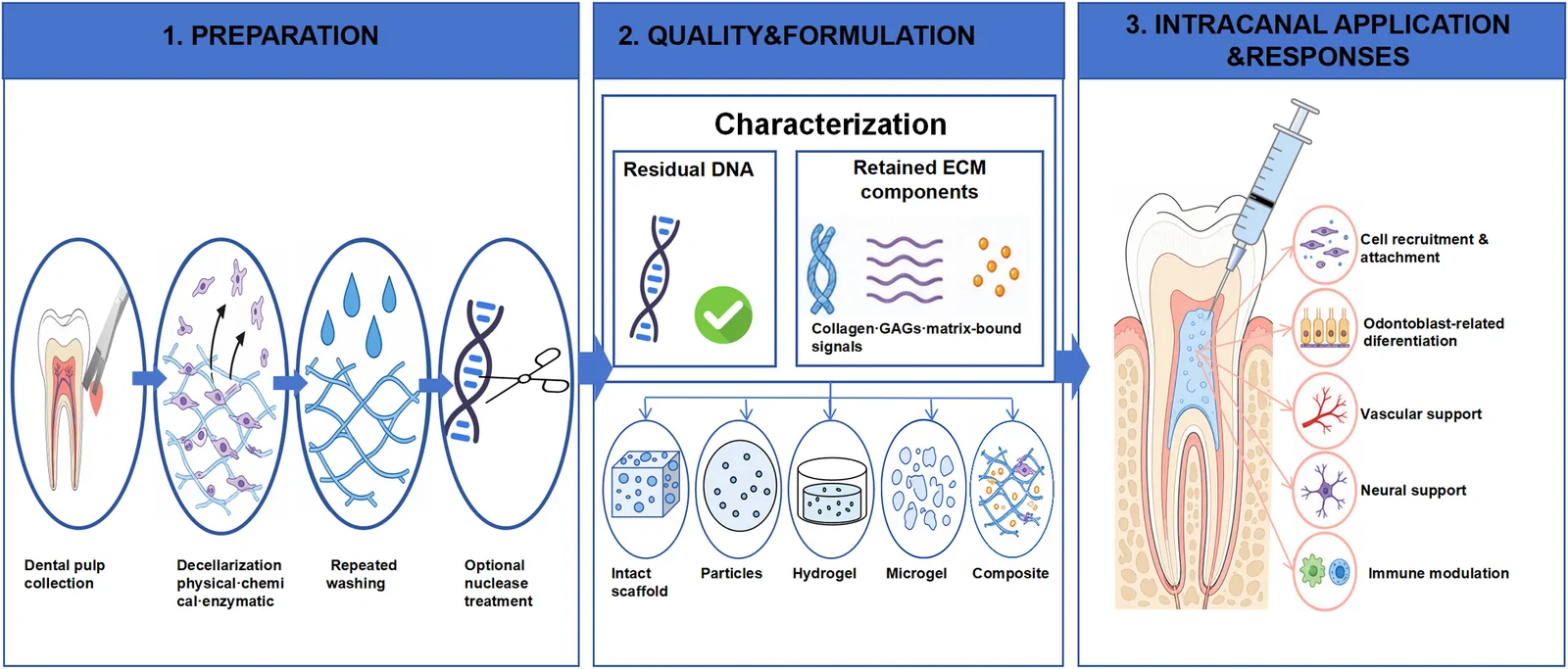
Complete preparation and application workflow for native tissue-derived DPECM. The scheme shows dental pulp collection, decellularization, repeated washing, optional nuclease treatment, sterilization, characterization of residual DNA and retained matrix components, processing into intact scaffolds, particulates, hydrogels, microgels, or composites, and final intracanal application. Any signaling, vascular, neural, immune, or odontoblast-related outcomes shown are proposed or preclinical and do not imply restored function or clinical efficacy.

### 1.1 Review scope and terminology

In this review, core DPECM refers only to decellularized extracellular matrix obtained directly from dental pulp tissue, whether retained as an intact scaffold or processed as a particulate, digest, or hydrogel. DPSC- or SHED-derived cell-deposited ECM is termed cell-derived dental ECM and is treated as related, not core, DPECM evidence. Matrices from bone, bladder, submandibular gland, amnion, periapical lesions, nerve, or tooth bud are comparative non-pulp dECMs. DPECM-containing microgels, cell- or extracellular-vesicle-loaded matrices, and photocrosslinked polymer hybrids are described as composite or combination systems; effects of the complete formulation are not attributed to DPECM alone. Terminology for outcomes is also used conservatively: repair denotes structural healing without recreation of native pulp; revitalization denotes re-establishment of living tissue within the canal without proof of native identity or function; pulp-like tissue formation is a histological description; and regeneration is reserved for evidence approaching restoration of organized pulp-dentin structure and function.

### 1.2 Review methods

PubMed was searched from database inception to 31 July 2026. The core strategy combined (“dental pulp” OR “pulp-dentin complex” OR “regenerative endodontics”) with (“decellularized extracellular matrix” OR “decellularized dental pulp matrix” OR DPECM OR dECM); supplementary searches added hydrogel, microgel, vascularization, innervation, macrophage, mechanotransduction, and extracellular vesicle terms. Reference lists of eligible articles were also screened. English-language peer-reviewed original studies and reviews were included when they directly informed DPECM source, processing, material properties, cell responses, mechanisms, delivery, preclinical outcomes, or clinical context; comparative non-pulp dECM studies were retained only when they clarified specificity or design. Duplicates, conference abstracts, non-peer-reviewed reports, and studies without relevant dental or comparator evidence were excluded. Records underwent title/abstract screening followed by full-text assessment, and 101 articles were retained for narrative synthesis. Primary research articles were prioritized for claims about biological activity, mechanisms, and regenerative outcomes; reviews were used mainly for background and evidence synthesis. The retained set encompassed all references cited in this review, including general background, methodological, and review articles; no citations were drawn from outside the screened literature library.

## 2 Preparation and material properties of DPECM

### 2.1 Tissue sources and decellularization methods

Bovine pulp can be converted into an injectable hydrogel while retaining collagen, glycosaminoglycans, and measurable amounts of TGF-*β*1, bFGF, BMP-2, and VEGF. Adding hyaluronic acid changes the pores, degradation behavior, and release profile (7). A canine study published in 2026 tested porcine DPECM hydrogel in an orthotopic model. It reported more pulp-like tissue formation than collagen or bleeding-based controls (8). Other porcine studies have reached a similar practical point: concentration matters, and injectability matters just as much (9). Human-source DPECM avoids xenogeneic source mismatch and has been prepared as both intact and solubilized scaffolds. Work with bone-derived dECM also shows that dental stem cells can respond to a non-pulp matrix when its composition and mechanics are suitable (10, 11). Even after these advances, recent systematic assessment found very few direct, well-controlled comparisons between processing protocols (12). The complete preparation workflow—from tissue collection through decellularization, washing, optional nuclease treatment, sterilization, characterization, formulation, and final application—is outlined in Figure 1.

The field has gradually moved away from intact scaffolds alone and toward materials that can actually be delivered through a root canal. Gelatin microspheres functionalized with DPECM can carry cells and bioactive factors as injectable microcarriers. They may also lessen the diffusion problems seen in large hydrogel volumes (13). As comparative, non-DPECM evidence, bladder-derived dECM has been used to study matrix-dependent communication between DPSCs and macrophages (14). Reviews centered on DPECM, meanwhile, have made the same point repeatedly: tissue-derived scaffolds offer biological advantages, but their manufacture is still difficult to standardize (15). Newer designs combine DPECM with built-in immune regulation, angiogenic-odontogenic coupling, Jagged1-conditioned cell matrix, secretome delivery, or photocrosslinkable GelMA (16–19). These additions give researchers more control. They also create extra questions about crosslinker exposure, residual chemicals, mechanics, release behavior, and potency. Other tissue sources are considered here only as comparators when assessing whether pulp matrix is uniquely instructive. Decellularized submandibular gland, DPSC-derived ECM, human amniotic membrane, and matrix from periapical lesions can each support some part of dental-cell attachment or regeneration (20–23). This suggests that several benefits may come from shared ECM functions, including hydration, adhesion, growth-factor binding, and immune modulation. A fair test of pulp specificity therefore needs head-to-head comparisons with matched protein concentration, stiffness, degradation rate, and delivery format.

*In vitro*, human DPECM hydrogel promotes DPSC migration and proliferation and supports odontogenic or neural-like differentiation. Whole human pulp scaffolds can be repopulated while keeping a recognizable tissue architecture (24, 25). Bovine hydrogel has been studied in a necrotic-tooth model. Porcine DPECM has also been compared directly with nerve-derived dECM through proteomic and transcriptomic analyses (26, 27). Feline implantation and BMP4-conditioned DPECM constructs, together with comparative evidence from SHED-derived cell-deposited ECM and macrophage-modulating hydrogels, point to the practical lesson that both the source matrix and the surrounding biological setting shape the tissue that develops (28–31). Systematic reviews still describe DPECM as promising, but they also stress the wide variation in protocols and the limited certainty of the evidence (32). Studies that focus on processing make it clear why a single DNA cutoff cannot define a successful decellularization method. Detergent-treated swine pulp scaffolds have supported regenerative responses (33). In bovine pulp, limited exposure to Triton X-100 and trypsin/EDTA, without SDS, retained more glycosaminoglycans than harsher protocols (34). Comparisons in human tissue found meaningful differences in histology and proteomic retention. Researchers have also tried extracellular-vesicle enrichment and laminin replacement to restore signals lost during processing (35–37). Cyclic lavage, mechanical stirring, and low-concentration SDS have been refined with quantitative proteomics (38). Pairing DPECM with treated dentin matrix or aligned polymer scaffolds may help rebuild organization at the tissue interface, and porcine tooth-pulp work supports feasibility in larger animals (39, 40). Decellularized tooth-bud ECM can even guide tooth-like development. That finding is interesting, but it should not be confused with regeneration of adult pulp (41). Representative DPECM sources, processing strategies, delivery formats, experimental models, and major biological findings are summarized in Table 1.

**Table 1:** Representative sources, formulations, and experimental uses of DPECM.

Study	Source and format	Model	Principal finding	Main limitation
Matoug-Elwerfelli et al. (25)	Human whole-pulp scaffold	*In vitro* recellularization	Kept the native architecture and allowed DPSCs to attach and remain viable.	The study did not test an orthotopic outcome.
Song et al. (10)	Human decellularized pulp	*In vitro* and ectopic	Provided early evidence that human DPECM could serve as a scaffold.	This was an early proof-of-concept study.
Alqahtani et al. (40)	Porcine whole-pulp DPECM	Dog root canal	Showed cell entry together with pulp and vascular markers.	The experiment was small and follow-up was short.
Bakhtiar et al. (34)	Bovine pulp; protocol comparison	*In vitro*/subcutaneous	Showed that processing conditions changed GAG retention and the host response.	No single protocol was shown to work best in every setting.
Li et al. (24)	Human solubilized DPECM hydrogel	*In vitro*	Improved migration, proliferation, and several differentiation responses.	It was not tested in a disease-relevant *in vivo* model.
Alghutaimel et al. (46)	Bovine DPECM + human DPSCs	*In vitro*/preliminary *in vivo*	Supported an angiogenic phenotype and vascular repopulation.	Perfusion and long-term durability were not established.
Zheng et al. (13)	DPECM-functionalized gelatin microspheres	Semi-orthotopic	Improved injectability and supported pulp-like and dentin-like tissue formation.	The model was cell-loaded and ectopic.
Liang et al. (27)	Porcine DPECM hydrogel	Multi-omics and transplantation	Identified tenascin-C/Notch as a candidate mechanism requiring independent replication.	The finding still needs independent replication.
Elnawam et al. (7)	Bovine DPECM +/- hyaluronic acid	*In vitro*	Retained and released several growth factors.	Composition and potency were examined only *in vitro*.
Liang et al. (8)	Porcine DPECM hydrogel	Orthotopic beagle model	Improved pulp-like, dentinogenic, vascular, and neural-marker outcomes.	Evidence was marker-based and limited to 90 days.
Yi et al. (16)	Immunomodulatory porcine DPECM hydrogel	Vital-pulp regeneration	Linked immune regulation with angiogenic and odontogenic repair.	The indication differed from full-canal regeneration.
Yang et al. (19)	GelMA-DPECM photo-crosslinked hydrogel	*In vitro*/ectopic	Improved structural stability while preserving biological activity.	Light delivery and photoinitiator safety remain unresolved.

## 3 Biological effects and candidate mechanisms of DPECM

### 3.1 Cell attachment, proliferation, and odontogenic differentiation

A material for regenerative endodontics has to support more than one cell population. Driving mineral deposition alone is not enough. Recent reviews instead emphasize the coordination among stem or progenitor cells, scaffold chemistry, and soluble signals (42). As related rather than native-DPECM evidence, DPSC-derived cell-deposited ECM has increased mineral deposition and osteogenic or odontogenic responses *in vitro* through pathways that include Hippo and Wnt signaling (43, 44). When cell-derived ECM proteins are incorporated into self-assembling colloidal microgels, the matrix signals remain accessible while the material stays injectable (45).

Most studies judge DPECM-induced differentiation by alkaline phosphatase activity, mineral deposition, and expression of DSPP, DMP1, RUNX2, or collagen I. These measurements are useful, but they do not show that a polarized odontoblast layer has formed or that it is producing organized tubular dentin. Future work should examine where cells sit at the pulp-dentin interface, how the new matrix is arranged, how fast mineral forms, and whether unwanted calcification narrows the canal. Controls also need matched stiffness and collagen concentration. Without that, a response attributed to DPECM may simply reflect substrate mechanics or total protein density.

### 3.2 Vascularization and neural support

Vascularization remains one of the main bottlenecks. Cells entering a long canal receive blood only through the narrow apical opening, so early vessel growth is critical. Bovine DPECM seeded with human DPSCs has supported vascular repopulation and expression of angiogenic markers (46). Comparative studies of non-DPECM hyaluronic-acid, antimicrobial GelMA, and other angiogenic hydrogels illustrate how pore size, degradation, and factor release might be optimized, but they do not establish DPECM-specific vascular effects (47–49). DPSCs may contribute through paracrine signaling and may also take on perivascular or endothelial-like features. Lineage tracing is needed before those two possibilities can be separated (50).

A regenerated pulp should also regain neural function. DPSCs have neurotrophic activity and can show neural differentiation when the scaffold provides suitable cues (51). Still, positive staining for *β*III-tubulin, MAP2, or nestin does not demonstrate restored sensation. A stronger assessment would combine nerve-fiber density and orientation with spatial analysis of nerves and vessels, electrophysiology, and standardized sensibility tests.

### 3.3 Immune regulation and extracellular vesicles

An infected canal, or even a canal that has just been disinfected, is very different from a healthy culture dish. Fragments released from dECM can affect macrophage recruitment and phenotype. Signals retained in, or added to, the matrix may then connect resolution of inflammation with angiogenesis and dentin repair. DPECM studies now examine macrophages directly, but they use different cell sources, activation methods, and definitions of M1 and M2 states. It would be more useful to follow cytokine programs over time and to measure phagocytosis, matrix turnover, and communication among macrophages, endothelial cells, and odontogenic progenitors.

Extracellular vesicles and exosomes carry concentrated mixtures of microRNAs, proteins, and lipids that can influence odontogenesis, angiogenesis, and immune regulation (52–54). In cell-homing strategies, these vesicles may recruit and instruct host cells without requiring transplantation of expanded cells (55). As combination-product and comparative evidence, exosome-loaded chitin hydrogels and extracellular-vesicle-loaded fibrin gels have supported sustained delivery and vascular-marker outcomes, while decellularized endothelial-cell ECM has promoted an endothelial-like phenotype in dental progenitors (56–58); these findings should not be interpreted as effects of native DPECM. These systems are attractive, but they are combination products in practice. Vesicle identity, dose, loading efficiency, release, and potency need to be controlled separately from the scaffold itself.

### 3.4 Candidate mechanisms and evidential limits

#### 3.4.1 Matrix adhesion and mechanotransduction

DPECM exposes cells to collagen, fibronectin, laminins, and matricellular proteins. Integrins on DPSCs, endothelial cells, fibroblasts, and immune cells bind these ligands. In general cell-matrix biology, integrin clustering can activate focal-adhesion kinase and downstream PI3K/AKT, and cytoskeletal signaling; this sequence remains a candidate mechanism in DPECM because direct pathway-perturbation evidence is sparse. The response depends on which ligands are accessible and on the matrix stiffness, viscoelasticity, and degradation rate. Solubilization may uncover peptides that were previously hidden. Crosslinking may keep the material in place longer, but it can also slow cell invasion. These established cell-matrix principles provide a biologically plausible framework, but direct causal evidence in DPECM systems remains limited; most studies report pathway activation or biomarker changes rather than ligand-specific perturbation and rescue.

Matrix mechanics are part of the biology, not a secondary engineering detail. YAP/TAZ-Hippo and Wnt/*β*-catenin involvement is supported mainly by *in vitro* work with DPSC-derived cell-deposited ECM and pathway inhibitors (43), rather than being established across tissue-derived DPECM products. The problem is that changing matrix concentration or crosslinking often changes ligand density at the same time. Experiments need to separate these variables. A single elastic-modulus value is also incomplete. Cells remodel the material over time, so time-dependent rheology and degradation should be measured as well.

#### 3.4.2 Growth-factor retention and tissue-specific signaling

Glycosaminoglycans and other matrix proteins bind members of the TGF-*β*, BMP, FGF, and VEGF families. Instead of receiving one short burst of soluble factor, nearby cells encounter a local reservoir. One comparative omics study identified tenascin-C as an abundant DPECM component and used Notch-pathway inhibition to support a possible tenascin-C/Notch contribution to odontoblast differentiation (27). This candidate mechanism is based on limited evidence, has not been independently replicated, and should not be regarded as an established or defining feature of DPECM. When decellularization removes too much basement-membrane information, replacing laminin can improve cell attachment and formation of an odontoblast-like layer (37). Jagged1-conditioned cell-derived ECM offers another example: a matrix can be deliberately prepared to present a selected signaling cue (17).

The difficult part is proving cause and effect. Omics associations need follow-up experiments that remove the candidate component, block its receptor, rescue the response, or rebuild the system with defined ingredients. Without those tests, an apparent pulp-specific effect might actually come from stiffness, residual soluble factors, or a different degradation rate. Mechanistic work should also include several donor lots and, where possible, more than one species. Only then can a proposed pathway be treated as a stable feature of the product.

#### 3.4.3 Coupling among immune, vascular, and odontogenic responses

Macrophages are among the first cells to enter an implanted matrix. Their behavior can shape endothelial organization, fibroblast activity, and odontogenic differentiation. Recent DPECM hydrogel studies report associations among macrophage phenotype, angiogenic or vascular markers, and dentin repair (16, 31), but the evidence is model-specific and does not yet establish a conserved causal sequence. This is closer to the clinical situation than studying odontogenesis alone. A previously infected canal may still contain bacterial products, inflammatory cytokines, hypoxic regions, and traces of irrigants. Disease-relevant models should therefore include controlled biofilm exposure and realistic disinfection before the matrix is placed. Extracellular vesicles introduce another regulatory layer because they transfer microRNAs and proteins (52–57). The matrix can act both as a physical niche and as a depot for vesicles. Yet vesicles may change during storage, and some may leave the target site after delivery. Calling such a system “cell-free” should not automatically imply low risk. Pharmacokinetic and biodistribution studies are still needed.

## 4 DPECM delivery systems and biofabrication

Because direct DPECM delivery studies remain limited, the following non-DPECM platforms are discussed only as design comparators for controlled signal delivery. Examples include silk-fibroin scaffolds carrying bFGF, three-dimensional printed alginate/gelatin constructs, and VEGF-loaded alginate/laponite microspheres (59–61). Notch-responsive hydrogels can speed vascularization. Carbon-dot-mediated photocrosslinking offers another way to stabilize GelMA-based materials (62, 63). Scaffold-free DPSC sheets have guided axonal growth, suggesting that aligned endogenous ECM can direct neurites even without an added carrier (64). Earlier work with collagen-like injectable hydrogels established a simpler but essential requirement: DPSCs must remain viable and able to spread during gelation (65, 66).

Prevascularization can be attempted before implantation by combining endothelial cells, pericyte-like cells, DPSCs, microchannels, or angiogenic factors (67). Umbilical-cord mesenchymal cells can express odontoblast-like and endothelial markers, although their use raises additional immune and regulatory concerns (68). DPSC-derived cell-deposited ECM is a distinct, potentially more reproducible cell-made matrix and is not interchangeable with native tissue-derived DPECM. In contrast, DPSC constructs prepared with platelet-rich fibrin contain many biological signals but can vary considerably from batch to batch (69, 70). The right design depends on the clinical problem. A cell-free hydrogel for an immature tooth does not need the same potency profile or release behavior as a cell-loaded material for a mature necrotic tooth.

## 5 Preclinical DPECM evidence and separate clinical context

### 5.1 What animal studies and histology actually show

A 2024 systematic review examined 17 animal studies of dECM scaffolds from multiple sources, not DPECM alone. These studies generally reported angiogenic markers and pulp-like or dentin-like tissue formation; they do not establish functional regeneration or a DPECM-specific effect. The studies were too different in matrix source, processing, cell loading, animal species, and endpoints for a reliable quantitative comparison, and the risk of bias was high (71). Human histology after REP often shows fibrous connective tissue, cementum-like tissue, or bone-like deposits rather than a reconstructed native pulp. Immunohistological work in infected immature teeth, together with reviews of animal and human tissue findings, warns against treating radiographic root development as proof of true regeneration (72–74).

Teeth removed after failed post-trauma treatment have provided rare human specimens. They show how strongly intracanal healing depends on infection control, the condition of apical tissues, and injury caused during the procedure (75). Some individual teeth contain vascular connective tissue and mineralized deposits after REP, but the cellular arrangement is inconsistent (76, 77). Electron microscopy performed 16 years after treatment confirmed that intracanal tissue can persist for a long time. It did not, however, reproduce normal pulp architecture (78). The same caution applies to mature teeth. Tissue may fill the canal, but routine radiographs cannot establish where it came from, what it is, or whether it functions as pulp (79).

### 5.2 Absence of direct clinical DPECM evidence and relevant clinical context

As of 31 July 2026, no published human clinical study was identified that directly evaluated a DPECM-based therapeutic product for pulp-dentin regeneration. The clinical studies summarized below concern cell therapy, conventional regenerative endodontic procedures, platelet-based scaffolds, imaging, or cell homing and are included only as translational context; they are not evidence of DPECM clinical efficacy. Cell-based regenerative endodontics has entered early clinical testing. A randomized phase I/II trial showed that cell treatment for periapical lesions was feasible. Magnetic resonance imaging has also been explored as a way to estimate intracanal tissue after REP in mature teeth (80, 81). Early randomized studies in mature necrotic teeth and observational studies in immature teeth report healing. Some also describe responses to sensibility tests or continued root growth (82–84). Leukocyte- and platelet-rich fibrin has been tested in multicenter protocols, and recent reviews describe a growing clinical literature on cell homing (85, 86).

Transplanting stem or progenitor cells may offer more regenerative capacity than relying on host-cell recruitment, particularly when few apical cells remain. That possible advantage comes with practical problems: the cells must be sourced, expanded, identified, delivered, and monitored for safety, including tumorigenic risk. Clinical perspectives and animal studies support further work with dental-pulp cells, bone-marrow stromal cells, and other mesenchymal populations (87–89). One report using allogeneic stromal cells in a mature tooth showed feasibility, but a single case cannot establish comparative benefit (90). For evidence synthesis, DPECM should therefore be considered separately as an acellular material and as a carrier for transplanted cells. Combining the two hides important differences in mechanism and risk. The evidence hierarchy for DPECM translation, with non-DPECM human studies explicitly separated as contextual evidence, is summarized in Table 2. Product-specific immune, manufacturing, quality-control, and regulatory considerations are compared in Table 3.

**Table 2:** Evidence hierarchy for DPECM translation and clearly separated non-DPECM clinical context.

Evidence level	What it can establish	Representative evidence	Common overinterpretation	Priority improvement
Material characterization	Product identity, remaining DNA, architecture, mechanics, and retained signals	Protocol comparisons and proteomics (7, 34, 35, 38)	Treating biochemical retention as proof of potency	Connect release criteria to a validated biological assay
*In vitro* cell assays	Cell compatibility, migration, and possible signaling pathways	DPECM hydrogels and related cell-derived ECM studied *in vitro* (24, 43–46)	Treating marker expression as functional regeneration	Use mechanics-matched controls and pathway perturbation
Ectopic implantation	Host-cell entry, degradation, and vascular compatibility	Whole matrices, microgels and hybrid hydrogels (13, 19, 25)	Calling subcutaneous pulp-like tissue evidence of clinical efficacy	Measure perfusion and extend the remodeling period
Semi-orthotopic tooth models	Responses to canal geometry, dentin, and interface signals	DPECM micros pheres and composite constructs (13, 39)	Leaving out infection, apical blood supply, and coronal sealing	Use standardized infected-root and irrigation models
Orthotopic large-animal models	Feasibility of delivery and tissue formation within the jaw	Porcine DPECM in dogs and 2026 canine hydrogel study (8, 40)	Describing marker-positive tissue as fully functional	Assess sensation, perfusion, reinfection, and long-term outcomes
Human clinical or histological context (no direct DPECM trial)	Contextual safety, healing, and biological outcomes of non-DPECM regenerative approaches	REP histology and early cell trials, included only as context (72–90)	Treating non-DPECM clinical outcomes as evidence of DPECM efficacy	Conduct first-in-human DPECM studies only after standardized preclinical safety and potency testing

**Table 3:** Product-specific translational risks, quality attributes, and regulatory considerations for DPECM-based products.

Product type	Principal translational risks	Critical quality controls	Likely regulatory implication	Priority evidence gap
Acellular DPECM hydrogel	Donor and lot variability; residual DNA, detergent, enzyme, endotoxin, or bioburden; loss of matrix potency after sterilization or storage.	Source traceability; identity and composition; dsDNA and residual-process testing; sterility/endotoxin; rheology, gelation, degradation, and intended-use potency.	Tissue-derived biomaterial; requirements depend on source and jurisdiction, with added scrutiny for allogeneic material.	Validated release criteria linked to durable orthotopic function.
Xenogeneic DPECM	Residual xenoantigens and immune reaction; zoonotic or adventitious-agent transmission; species-dependent composition.	Species and herd traceability; antigen-removal evidence; viral/adventitious-agent testing; immunogenicity and lot comparability.	Higher biological-safety burden than human-source acellular matrix.	Long-term immune and reinfection studies in clinically relevant root models.
DPECM microgels or microspheres	Particle-size variability, syringe blockage, uneven canal filling, altered degradation, and carrier-related effects.	Particle-size distribution; injectability; carrier identity; payload uniformity; swelling, retention, degradation, and sterility.	May be treated as a biomaterial-device or combination product, depending on carrier and payload.	Delivery accuracy, apical containment, and performance in irregular mature canals.
DPECM combined with cells	Cell identity and heterogeneity; contamination; immune incompatibility; ectopic differentiation, calcification, or tumorigenic risk; cold-chain complexity.	GMP cell sourcing and expansion; identity, purity, viability, dose, sterility, genomic stability, and combined-product potency.	Cell-based combination product with substantially greater manufacturing and clinical-monitoring requirements.	Comparative benefit over cell-free DPECM and long-term safety.
DPECM combined with extracellular vesicles	Variable vesicle identity, cargo, dose, loading and release; aggregation, storage instability, off-target distribution, and unclear pharmacokinetics.	Vesicle identity and purity; particle and protein dose; loading efficiency; release; stability; biodistribution; scaffold and vesicle potency assays.	Biologic-combination product; scaffold and vesicle components require separate and joint characterization.	Dose-response, biodistribution, persistence, and reproducible potency.
Photocrosslinked DPECM composites	Residual monomer or photoinitiator; cytotoxicity; nonuniform cure depth; light-delivery constraints; stiffness and degradation changes.	Composition and residuals; light dose and cure depth; conversion; mechanics, swelling, degradation, leachables, sterility, and biocompatibility.	Biomaterial-device or combination pathway may apply; the crosslinking system adds safety and process controls.	Intracanal curing feasibility and chronic local safety after realistic irrigation.

## 6 Discussion and future directions

DPECM research began with basic questions about cell compatibility. It has since moved toward injectable materials, immune-responsive designs, and matrices built around selected signaling pathways. Reviews covering both laboratory studies and clinical regenerative endodontics point to an uncomfortable gap: the biomaterials are becoming more sophisticated, but routine clinical outcomes still do not reliably reproduce native pulp biology (91). Recent clinical datasets confirm that REP can support apical healing and continued root development. Intracanal calcification and uncertain tissue identity remain common concerns (92). Some newer strategies combine acellular pulp matrix with allogeneic cells. Others move from simple hydrogels to microgels, bioinks, or spatially patterned constructs (93, 94). These approaches should be judged by the problem they solve, not ranked by how technically complex they look (95, 96). Developmentally primed odontogenic exosomes have been associated with extensive pulp-dentin-like tissue formation *in vivo*, supporting further study of ECM as a vesicle depot without establishing functional or comparative superiority (97).

The first practical priority is manufacturing consistency. Source species, donor age and health, time before processing, tissue pooling, detergent exposure, nuclease treatment, digestion, crosslinking, sterilization, and storage all need to be controlled. Release testing should cover product identity, dsDNA amount and fragment size, residual detergents and enzymes, endotoxin, mycoplasma, sterility, viral risk, rheology, gelation, degradation, and a potency assay tied to the intended use. Sterilization should not be judged only by whether microorganisms are killed. Gamma irradiation, ethylene oxide, peracetic acid, and supercritical carbon dioxide can alter matrix structure and signaling in different ways. The second priority is testing under conditions that resemble treatment. A scaffold must pass through a narrow needle, fill an irregular canal without leaving voids, remain below the cervical barrier, and gel within a workable time. It then has to function after exposure to sodium hypochlorite, EDTA, intracanal medicaments, and residual microbial products. Most studies still use healthy dentin and do not model reinfection. Standardized infected-root models, realistic irrigation, and reliable coronal sealing would provide a better bridge to large-animal studies. These source-specific and standardization considerations parallel those identified for natural biomaterials in osteochondral repair, where material origin, processing, quality control, and evaluation frameworks shape the path from biological promise to clinical translation (98).

Outcome definitions also need to become more demanding. Cell density and marker staining are not enough to claim a functional pulp. Perfused microvessels, organized sensory nerves, controlled tubular dentin formation, immune responsiveness, absence of obstructive calcification, and resistance to reinfection describe different parts of the same outcome. Routine histology will rarely be possible in human trials. A core outcome set should therefore combine validated imaging, sensibility testing, biomarkers, and tissue analysis when treated teeth are later extracted. Reviews of biological scaffolds and transplantation models show that current reporting is still too inconsistent for confident comparison (99, 100). Added complexity should solve a clearly demonstrated problem. Exosomes, laminin, antimicrobial peptides, photocrosslinking, and microcarriers may each address a real limitation, but every added element makes manufacturing and regulation harder. Early clinical experience with dental-tissue-derived cell products shows that translation is possible when cell identity, safety, and delivery are tightly controlled (101). For a defined group of patients, the most useful DPECM product may turn out to be a fairly simple, standardized, cell-free hydrogel rather than the formulation with the longest ingredient list.

This review has limitations. The search was focused and was not registered as a systematic review. Study heterogeneity did not allow a meta-analysis, and several papers published in 2026 have not yet been independently reproduced. Even with these limitations, the literature supports tissue-engineering potential for DPECM, but the present level of certainty remains predominantly preclinical and moderate to low. The field now needs better comparability, clearer mechanisms, controlled manufacturing, and evidence of durable function more than it needs another slightly different formulation.

## 7 Conclusion

DPECM carries structural and biochemical information that is difficult to reproduce with purified materials. Whole matrices keep more of the native architecture. Hydrogels, microspheres, and hybrid composites are easier to deliver through a root canal, although processing changes the original matrix. Cell and animal studies indicate that DPECM can support cell recruitment, odontogenic marker expression, vascular-marker expression or vessel-like structures, and neural-marker expression. Orthotopic studies strengthen the preclinical rationale for pulp-derived matrix but do not demonstrate restored perfusion, sensation, organized tubular dentin, or clinical superiority. The evidence is not yet strong enough to prove clinical effectiveness because tissue sources, processing methods, controls, and outcomes vary widely, and no robust human comparative evidence establishes that DPECM is superior to current regenerative procedures. Before routine clinical use, the field needs well-defined products, causally tested mechanisms, disease-relevant models, and durable functional outcomes.

## References

[1] Kim SG Malek M Sigurdsson A Lin LM Kahler B. Regenerative endodontics: a comprehensive review. Int Endod J. (2018) 51(12):1367–88. 10.1111/iej.12954 29777616

[2] Galler KM D’Souza RN Hartgerink JD Schmalz G. Scaffolds for dental pulp tissue engineering. Adv Dent Res. (2011) 23(3):333–9. 10.1177/0022034511405326 21677088

[3] Galler KM Eidt A Schmalz G. Cell-free approaches for dental pulp tissue engineering. J Endod. (2014) 40(4 Suppl):S41–5. 10.1016/j.joen.2014.01.014 24698692

[4] Gilbert TW Sellaro TL Badylak SF. Decellularization of tissues and organs. Biomaterials. (2006) 27(19):3675–83. 10.1016/j.biomaterials.2006.02.014 16519932

[5] Badylak SF Freytes DO Gilbert TW. Extracellular matrix as a biological scaffold material: structure and function. Acta Biomater. (2009) 5(1):1–13. 10.1016/j.actbio.2008.09.013 18938117

[6] Crapo PM Gilbert TW Badylak SF. An overview of tissue and whole organ decellularization processes. Biomaterials. (2011) 32(12):3233–43. 10.1016/j.biomaterials.2011.01.057 21296410 PMC3084613

[7] Elnawam H Thabet A Mobarak A Abdallah A Elbackly R. Preparation and characterization of bovine dental pulp-derived extracellular matrix hydrogel for regenerative endodontic applications: an *in vitro* study. BMC Oral Health. (2024) 24(1):1281. 10.1186/s12903-024-05004-z 39448989 PMC11515367

[8] Liang Z Feng Y Zhang S Li J Rao Z Zhang K. Decellularized dental pulp matrix hydrogel promotes functional endodontic regeneration in situ. Int Endod J. (2026) 59(8):1680–93. 10.1111/iej.70148 41872666 PMC13373075

[9] Yuan S Yang X Wang X Chen J Tian W Yang B. Injectable xenogeneic dental pulp decellularized extracellular matrix hydrogel promotes functional dental pulp regeneration. Int J Mol Sci. (2023) 24(24):17483. 10.3390/ijms242417483 38139310 PMC10743504

[10] Song JS Takimoto K Jeon M Vadakekalam J Ruparel NB Diogenes A. Decellularized human dental pulp as a scaffold for regenerative endodontics. J Dent Res. (2017) 96(6):640–6. 10.1177/0022034517693606 28196330

[11] Paduano F Marrelli M White LJ Shakesheff KM Tatullo M. Odontogenic differentiation of human dental pulp stem cells on hydrogel scaffolds derived from decellularized bone extracellular matrix and collagen type I. PLoS One. (2016) 11(2):e0148225. 10.1371/journal.pone.0148225 26882351 PMC4755593

[12] Saith H Shetty S Nireeksha LJ. Cellular and molecular response of dental stem cells to decellularized extracellular matrix scaffolds in regenerative endodontics: a systematic review. Front Dent Med. (2026) 7:1766825. 10.3389/fdmed.2026.1766825 41867637 PMC13002784

[13] Zheng L Liu Y Jiang L Wang X Chen Y Li L. Injectable decellularized dental pulp matrix-functionalized hydrogel microspheres for endodontic regeneration. Acta Biomater. (2023) 156:37–48. 10.1016/j.actbio.2022.11.047 36455855

[14] Nguyen H Kao C Chiang C Hung Y Lo C. Investigating the immunomodulatory potential of dental pulp stem cell cultured on decellularized bladder hydrogel towards macrophage response *in vitro*. Gels. (2022) 8(3):187. 10.3390/gels8030187 35323300 PMC8954673

[15] Khurshid Z Alnaim AJA Alhashim AAA Imran E Adanir N. Future of decellularized dental pulp matrix in regenerative endodontics. Eur J Dent. (2022) 16(4):737–41. 10.1055/s-0041-1741012 34991166 PMC9683870

[16] Yi P Zeng R Gao Z Zhao Y Li P Tan J. An immunomodulatory decellularized pulp matrix hydrogel promotes vascularized dental pulp regeneration through angiogenic-odontogenic coupling. Regen Biomater. (2026) 13:rbag107. 10.1093/rb/rbag107 42344368 PMC13287997

[17] Phothichailert S Nowwarote N Fournier BPJ Trachoo V Roytrakul S Namangkalakul W. Effects of decellularized extracellular matrix derived from Jagged1-treated human dental pulp stem cells on biological responses of stem cells isolated from apical papilla. Front Cell Dev Biol. (2022) 10:948812. 10.3389/fcell.2022.948812 36081912 PMC9445441

[18] Carvalho GL Sarra G Schröter GT Silva LSRG Ariga SKK Gonçalves F. Pro-angiogenic potential of a functionalized hydrogel scaffold as a secretome delivery platform: an innovative strategy for cell homing-based dental pulp tissue engineering. J Tissue Eng Regen Med. (2022) 16(5):472–83. 10.1002/term.3294 35244346

[19] Yang Y Zhao J Qin B Xu Z Wu L Shi W. Bioactive photo-crosslinkable hydrogel based on decellularized dental pulp matrix for functional pulp regeneration. BMC Oral Health. (2026) 26(1):654. 10.1186/s12903-026-08001-6 41787338 PMC13072613

[20] Shi Y Wang Y Shan Z Gao Z. Decellularized rat submandibular gland as an alternative scaffold for dental pulp regeneration. Front Bioeng Biotechnol. (2023) 11:1148532. 10.3389/fbioe.2023.1148532 37152652 PMC10160494

[21] Nowwarote N Chahlaoui Z Petit S Duong LT Dingli F Loew D. Decellularized extracellular matrix derived from dental pulp stem cells promotes gingival fibroblast adhesion and migration. BMC Oral Health. (2024) 24(1):1166. 10.1186/s12903-024-04882-7 39354504 PMC11443845

[22] Bakhtiar H Ashoori A Rajabi S Pezeshki-Modaress M Ayati A Mousavi MR. Human amniotic membrane extracellular matrix scaffold for dental pulp regeneration *in vitro* and *in vivo*. Int Endod J. (2022) 55(4):374–90. 10.1111/iej.13675 34923640

[23] Hu N Jiang R Deng Y Li W Jiang W Xu N. Periapical lesion-derived decellularized extracellular matrix as a potential solution for regenerative endodontics. Regen Biomater. (2024) 11:rbae050. 10.1093/rb/rbae050 38872841 PMC11170217

[24] Li J Rao Z Zhao Y Xu Y Chen L Shen Z. A decellularized matrix hydrogel derived from human dental pulp promotes dental pulp stem cell proliferation, migration, and induced multidirectional differentiation *in vitro*. J Endod. (2020) 46(10):1438–1447.e5. 10.1016/j.joen.2020.07.008 32679242

[25] Matoug-Elwerfelli M Duggal MS Nazzal H Esteves F Raïf E. A biocompatible decellularized pulp scaffold for regenerative endodontics. Int Endod J. (2018) 51(6):663–73. 10.1111/iej.12882 29197101

[26] Elnawam H Thabet A Mobarak A Khalil NM Abdallah A Nouh S. Bovine pulp extracellular matrix hydrogel for regenerative endodontic applications: *in vitro* characterization and *in vivo* analysis in a necrotic tooth model. Head Face Med. (2024) 20(1):61. 10.1186/s13005-024-00460-y 39438876 PMC11494807

[27] Liang Z Li J Lin H Zhang S Liu F Rao Z. Understanding the multi-functionality and tissue-specificity of decellularized dental pulp matrix hydrogels for endodontic regeneration. Acta Biomater. (2024) 181:202–21. 10.1016/j.actbio.2024.04.040 38692468

[28] Tan YY Abdullah D Abu Kasim NH Yazid F Mahamad Apandi NI Ramanathan A. Histological characterization of pulp regeneration using decellularized human dental pulp and mesenchymal stem cells in a feline model. Tissue Cell. (2024) 90:102484. 10.1016/j.tice.2024.102484 39068688

[29] Tan Q Cao Y Zheng X Peng M Huang E Wang J. BMP4-regulated human dental pulp stromal cells promote pulp-like tissue regeneration in a decellularized dental pulp matrix scaffold. Odontology. (2021) 109(4):895–903. 10.1007/s10266-021-00620-5 34086131

[30] Yang N Shen R Yang W Zhang S Gong T Liu Y. Biomimetic pulp scaffolds prepared from extracellular matrix derived from stem cells from human exfoliated deciduous teeth promote pulp-dentine complex regeneration. Int Endod J. (2024) 57(9):1279–92. 10.1111/iej.14099 38828966

[31] Yi P Chen S Zhao Y Ku W Lu H Yu D. An injectable dental pulp-derived decellularized matrix hydrogel promotes dentin repair through modulation of macrophage response. Biomater Adv. (2024) 161:213883. 10.1016/j.bioadv.2024.213883 38762928

[32] Adanir N Khurshid Z Ratnayake J. The regenerative potential of decellularized dental pulp extracellular matrix: a systematic review. Materials (Basel). (2022) 15(18):6386. 10.3390/ma15186386 36143698 PMC9505725

[33] Hu L Gao Z Xu J Zhu Z Fan Z Zhang C. Decellularized swine dental pulp as a bioscaffold for pulp regeneration. Biomed Res Int. (2017) 2017:9342714. 10.1155/2017/9342714 29387727 PMC5745671

[34] Bakhtiar H Rajabi S Pezeshki-Modaress M Ellini MR Panahinia M Alijani S. Optimizing methods for bovine dental pulp decellularization. J Endod. (2021) 47(1):62–8. 10.1016/j.joen.2020.08.027 33049226

[35] Ribeiro VMS Sousa MGC Garcia PR Santos LS Duarte ECB Corrêa JR. Histologic and proteomic profile of two methods to decellularize human dental pulp tissue. Arch Oral Biol. (2024) 157:105847. 10.1016/j.archoralbio.2023.105847 38000129

[36] Diomede F Fonticoli L Marconi GD Della Rocca Y Rajan TS Trubiani O. Decellularized dental pulp, extracellular vesicles, and 5-azacytidine: a new tool for endodontic regeneration. Biomedicines. (2022) 10(2):403. 10.3390/biomedicines10020403 35203612 PMC8962372

[37] Fu J Chen J Li W Yang X Yang J Quan H. Laminin-modified dental pulp extracellular matrix for dental pulp regeneration. Front Bioeng Biotechnol. (2020) 8:595096. 10.3389/fbioe.2020.595096 33520954 PMC7838611

[38] Zhang Z Bi F Huang Y Guo W. Construction of dental pulp decellularized matrix by cyclic lavation combined with mechanical stirring and its proteomic analysis. Biomed Mater. (2024) 19(4):045002. 10.1088/1748-605X/ad4245 38653259

[39] Chen G Chen J Yang B Li L Luo X Zhang X. Combination of aligned PLGA/gelatin electrospun sheets, native dental pulp extracellular matrix and treated dentin matrix as substrates for tooth root regeneration. Biomaterials. (2015) 52:56–70. 10.1016/j.biomaterials.2015.02.011 25818413

[40] Alqahtani Q Zaky SH Patil A Beniash E Ray H Sfeir C. Decellularized swine dental pulp tissue for regenerative root canal therapy. J Dent Res. (2018) 97(13):1460–7. 10.1177/0022034518785124 30067420

[41] Zhang W Yelick PC. *In vivo* bioengineered tooth formation using decellularized tooth bud extracellular matrix scaffolds. Stem Cells Transl Med. (2025) 14(2):szae076. 10.1093/stcltm/szae076 39729491 PMC11878782

[42] Farjaminejad R Farjaminejad S Garcia-Godoy F. Regenerative endodontic therapies: harnessing stem cells, scaffolds, and growth factors. Polymers (Basel). (2025) 17(11):1475. 10.3390/polym17111475 40508718 PMC12157830

[43] Kornsuthisopon C Nowwarote N Chansaenroj A Photichailert S Rochanavibhata S Klincumhom N. Human dental pulp stem cells derived extracellular matrix promotes mineralization via hippo and wnt pathways. Sci Rep. (2024) 14(1):6777. 10.1038/s41598-024-56845-1 38514682 PMC10957957

[44] Nowwarote N Petit S Ferre FC Dingli F Laigle V Loew D. Extracellular matrix derived from dental pulp stem cells promotes mineralization. Front Bioeng Biotechnol. (2021) 9:740712. 10.3389/fbioe.2021.740712 35155398 PMC8829122

[45] Aksel H Sarkar D Lin MH Buck A Huang GT. Cell-derived extracellular matrix proteins in colloidal microgel as a self-assembly hydrogel for regenerative endodontics. J Endod. (2022) 48(4):527–34. 10.1016/j.joen.2022.01.011 35077752

[46] Alghutaimel H Yang X Drummond B Nazzal H Duggal M Raïf E. Investigating the vascularization capacity of a decellularized dental pulp matrix seeded with human dental pulp stem cells: *in vitro* and preliminary *in vivo* evaluations. Int Endod J. (2021) 54(8):1300–16. 10.1111/iej.13510 33709438

[47] AlHowaish NA AlSudani DI AlMuraikhi NA. Evaluation of a hyaluronic acid hydrogel (restylane lyft) as a scaffold for dental pulp regeneration in a regenerative endodontic organotype model. Odontology. (2022) 110(4):726–34. 10.1007/s10266-022-00710-y 35471745

[48] Dubey N Ribeiro JS Zhang Z Xu J Ferreira JA Qu L. Gelatin methacryloyl hydrogel as an injectable scaffold with multi-therapeutic effects to promote antimicrobial disinfection and angiogenesis for regenerative endodontics. J Mater Chem B. (2023) 11(17):3823–35. 10.1039/d2tb02788g 36946228 PMC10160005

[49] Siddiqui Z Sarkar B Kim K-K Kadincesme N Paul R Kumar A. Angiogenic hydrogels for dental pulp revascularization. Acta Biomater. (2021) 126:109–18. 10.1016/j.actbio.2021.03.001 33689817 PMC8096688

[50] Tang MQ Ye L Gao B. Role of dental pulp stem cells to promote angiogenesis in cell-based regenerative endodontics. Chin J Dent Res. (2023) 26(3):129–42. 10.3290/j.cjdr.b4330823 37732679

[51] Lambrichts I Driesen RB Dillen Y Gervois P Ratajczak J Vangansewinkel T. Dental pulp stem cells: their potential in reinnervation and angiogenesis by using scaffolds. J Endod. (2017) 43(9S):S12–6. 10.1016/j.joen.2017.06.001 28781091

[52] Kong Q Wang Y Jiang N Wang Y Wang R Hu X. Exosomes as promising therapeutic tools for regenerative endodontic therapy. Biomolecules. (2024) 14(3):330. 10.3390/biom14030330 38540750 PMC10967740

[53] Ahmad P Estrin N Farshidfar N Zhang Y Miron RJ. Mechanistic insights into dental stem cells-derived exosomes in regenerative endodontics. Int Endod J. (2025) 58(9):1384–407. 10.1111/iej.14269 40497413 PMC12339802

[54] Ivica A Zehnder M Weber FE. Therapeutic potential of mesenchymal stem cell-derived extracellular vesicles in regenerative endodontics. Eur Cell Mater. (2021) 41:233–44. 10.22203/eCM.v041a17 33660242

[55] Kim D Kim SG. Cell homing strategies in regenerative endodontic therapy. Cells. (2025) 14(3):201. 10.3390/cells14030201 39936992 PMC11817319

[56] Wang S Xing X Peng W Huang C Du Y Yang H. Fabrication of an exosome-loaded thermosensitive chitin-based hydrogel for dental pulp regeneration. J Mater Chem B. (2023) 11(7):1580–90. 10.1039/d2tb02073d 36722937

[57] Zhang S Thiebes AL Kreimendahl F Ruetten S Buhl EM Wolf M. Extracellular vesicles-loaded fibrin gel supports rapid neovascularization for dental pulp regeneration. Int J Mol Sci. (2020) 21(12):4226. 10.3390/ijms21124226 32545804 PMC7352754

[58] Gong T Heng BC Xu J Zhu S Yuan C Lo ECM. Decellularized extracellular matrix of human umbilical vein endothelial cells promotes endothelial differentiation of stem cells from exfoliated deciduous teeth. J Biomed Mater Res A. (2017) 105(4):1083–93. 10.1002/jbm.a.36003 28076902

[59] Yang J Zhang Y Sun Z Song G Chen Z. Dental pulp tissue engineering with bFGF-incorporated silk fibroin scaffolds. J Biomater Appl. (2015) 30(2):221–9. 10.1177/0885328215577296 25791684

[60] Yu H Zhang X Song W Pan T Wang H Ning T. Effects of 3-dimensional bioprinting alginate/gelatin hydrogel scaffold extract on proliferation and differentiation of human dental pulp stem cells. J Endod. (2019) 45(6):706–15. 10.1016/j.joen.2019.03.004 31056297

[61] Zhang R Xie L Wu H Yang T Zhang Q Tian Y. Alginate/laponite hydrogel microspheres co-encapsulating dental pulp stem cells and VEGF for endodontic regeneration. Acta Biomater. (2020) 113:305–16. 10.1016/j.actbio.2020.07.012 32663663

[62] Zhang S Yu M Li M He M Xie L Huo F. Notch signaling hydrogels enable rapid vascularization and promote dental pulp tissue regeneration. Adv Sci (Weinh). (2024) 11(35):e2310285. 10.1002/advs.202310285 39013081 PMC11425206

[63] Cao Y Yang M Zhang R Ning X Zong M Liu X. Carbon dot-based photo-cross-linked gelatin methacryloyl hydrogel enables dental pulp regeneration: a preliminary study. ACS Appl Mater Interfaces. (2024) 16(18):22976–88. 10.1021/acsami.4c03168 38657655

[64] Drewry MD Dailey MT Rothermund K Backman C Dahl KN Syed-Picard FN. Promoting and orienting axon extension using scaffold-free dental pulp stem cell sheets. ACS Biomater Sci Eng. (2022) 8(2):814–25. 10.1021/acsbiomaterials.1c01517 34982537 PMC9821555

[65] Cavalcanti BN Zeitlin BD Nör JE. A hydrogel scaffold that maintains viability and supports differentiation of dental pulp stem cells. Dent Mater. (2013) 29(1):97–102. 10.1016/j.dental.2012.08.002 22901827 PMC3515741

[66] Jones TD Kefi A Sun S Cho M Alapati SB. An optimized injectable hydrogel scaffold supports human dental pulp stem cell viability and spreading. Adv Med. (2016) 2016:7363579. 10.1155/2016/7363579 27294191 PMC4884792

[67] Ruan Q Tan S Guo L Ma D Wen J. Prevascularization techniques for dental pulp regeneration: potential cell sources, intercellular communication and construction strategies. Front Bioeng Biotechnol. (2023) 11:1186030. 10.3389/fbioe.2023.1186030 37274160 PMC10232868

[68] Zhang S Zhang W Li Y Ren L Deng H Yin X. Human umbilical cord mesenchymal stem cell differentiation into odontoblast-like cells and endothelial cells: a potential cell source for dental pulp tissue engineering. Front Physiol. (2020) 11:593. 10.3389/fphys.2020.00593 32714196 PMC7344301

[69] Ravindran S Huang C George A. Extracellular matrix of dental pulp stem cells: applications in pulp tissue engineering using somatic MSCs. Front Physiol. (2014) 4:395. 10.3389/fphys.2013.00395 24432005 PMC3880843

[70] Chen Y-J Zhao Y-H Zhao Y-J Liu N-X Lv X Li Q. Potential dental pulp revascularization and odonto-/osteogenic capacity of a novel transplant combined with dental pulp stem cells and platelet-rich fibrin. Cell Tissue Res. (2015) 361(2):439–55. 10.1007/s00441-015-2125-8 25797716

[71] Elnawam H Abdallah A Nouh S Khalil NM Elbackly R. Influence of extracellular matrix scaffolds on histological outcomes of regenerative endodontics in experimental animal models: a systematic review. BMC Oral Health. (2024) 24(1):511. 10.1186/s12903-024-04266-x 38689279 PMC11061952

[72] Meschi N Hilkens P Lambrichts I Van den Eynde K Mavridou A Strijbos O. Regenerative endodontic procedure of an infected immature permanent human tooth: an immunohistological study. Clin Oral Investig. (2016) 20(4):807–14. 10.1007/s00784-015-1555-8 26250796

[73] Altaii M Richards L Rossi-Fedele G. Histological assessment of regenerative endodontic treatment in animal studies with different scaffolds: a systematic review. Dent Traumatol. (2017) 33(4):235–44. 10.1111/edt.12338 28342218

[74] Digka A Sakka D Lyroudia K. Histological assessment of human regenerative endodontic procedures (REP) of immature permanent teeth with necrotic pulp/apical periodontitis: a systematic review. Aust Endod J. (2020) 46(1):140–53. 10.1111/aej.12371 31432612

[75] Meschi N Hilkens P Van Gorp G Strijbos O Mavridou A Cadenas de Llano Perula M. Regenerative endodontic procedures posttrauma: immunohistologic analysis of a retrospective series of failed cases. J Endod. (2019) 45(4):427–34. 10.1016/j.joen.2019.01.007 30833096

[76] Lei L Chen Y Zhou R Huang X Cai Z. Histologic and immunohistochemical findings of a human immature permanent tooth with apical periodontitis after regenerative endodontic treatment. J Endod. (2015) 41(7):1172–9. 10.1016/j.joen.2015.03.012 25931029

[77] Nosrat A Kolahdouzan A Khatibi AH Verma P Jamshidi D Nevins AJ. Clinical, radiographic, and histologic outcome of regenerative endodontic treatment in human teeth using a novel collagen-hydroxyapatite scaffold. J Endod. (2019) 45(2):136–43. 10.1016/j.joen.2018.10.012 30711168

[78] Kwon S Kyeong M Adasooriya D Cho S Jung I. Histologic and electron microscopic characterization of a human immature permanent premolar with chronic apical abscess 16 years after regenerative endodontic procedures. J Endod. (2023) 49(8):1051–7. 10.1016/j.joen.2023.05.017 37268290

[79] Arslan H Şahin Y Topçuoğlu HS Gündoğdu B. Histologic evaluation of regenerated tissues in the pulp spaces of teeth with mature roots at the time of the regenerative endodontic procedures. J Endod. (2019) 45(11):1384–9. 10.1016/j.joen.2019.07.016 31515046

[80] Brizuela C Meza G Urrejola D Quezada MA Concha G Ramírez V. Cell-based regenerative endodontics for treatment of periapical lesions: a randomized, controlled phase I/II clinical trial. J Dent Res. (2020) 99(5):523–9. 10.1177/0022034520913242 32202965

[81] El-Kateb NM El-Backly RN Amin WM Abdalla AM. Quantitative assessment of intracanal regenerated tissues after regenerative endodontic procedures in mature teeth using magnetic resonance imaging: a randomized controlled clinical trial. J Endod. (2020) 46(5):563–74. 10.1016/j.joen.2020.01.026 32173020

[82] Youssef A Ali M ElBolok A Hassan R. Regenerative endodontic procedures for the treatment of necrotic mature teeth: a preliminary randomized clinical trial. Int Endod J. (2022) 55(4):334–46. 10.1111/iej.13681 35030270

[83] Diogenes A Ruparel NB. Regenerative endodontic procedures: clinical outcomes. Dent Clin North Am. (2017) 61(1):111–25. 10.1016/j.cden.2016.08.004 27912813

[84] Chrepa V Joon R Austah O Diogenes A Hargreaves KM Ezeldeen M. Clinical outcomes of immature teeth treated with regenerative endodontic procedures-A san antonio study. J Endod. (2020) 46(8):1074–84. 10.1016/j.joen.2020.04.008 32560972

[85] Meschi N EzEldeen M Garcia AET Lahoud P Van Gorp G Coucke W. Regenerative endodontic procedure of immature permanent teeth with leukocyte and platelet-rich fibrin: a multicenter controlled clinical trial. J Endod. (2021) 47(11):1729–50. 10.1016/j.joen.2021.08.003 34400199

[86] Yan H De Deus G Kristoffersen IM Wiig E Reseland JE Johnsen GF. Regenerative endodontics by cell homing: a review of recent clinical trials. J Endod. (2023) 49(1):4–17. 10.1016/j.joen.2022.09.008 36270575

[87] Shamszadeh S Eghbal MJ Asgary S. Advancing dentin-pulp regeneration: clinical perspectives and insights from stem/progenitor cell transplantation (part II). Am J Stem Cells. (2024) 13(3):132–42. 10.62347/BYPG4014 39021371 PMC11249670

[88] Raudales JLM Okuwa Y Honda M. Dental pulp cell transplantation combined with regenerative endodontic procedures promotes dentin matrix formation in mature mouse molars. Cells. (2024) 13(4):348. 10.3390/cells13040348 38391961 PMC10886544

[89] Gomez-Sosa JF Cardier JE Wittig O Díaz-Solano D Lara E Duque K. Allogeneic bone marrow mesenchymal stromal cell transplantation induces dentin pulp complex-like formation in immature teeth with pulp necrosis and apical periodontitis. J Endod. (2024) 50(4):483–92. 10.1016/j.joen.2024.01.002 38237659

[90] Gomez-Sosa JF Diaz-Solano D Wittig O Cardier JE. Dental pulp regeneration induced by allogenic mesenchymal stromal cell transplantation in a mature tooth: a case report. J Endod. (2022) 48(6):736–40. 10.1016/j.joen.2022.03.002 35307516

[91] Shi X Hu X Jiang N Mao J. Regenerative endodontic therapy: from laboratory bench to clinical practice. J Adv Res. (2025) 72:229–63. 10.1016/j.jare.2024.07.001 38969092 PMC12147620

[92] Song M Jung HI Kim SG. Clinical outcomes of regenerative endodontic procedure: periapical healing, root development, and intracanal calcification. J Endod. (2025) 51(6):722–31. 10.1016/j.joen.2025.02.007 39986569

[93] Mohd Noor NS Lam X-J Lim G-S Lau M-N Wey M-C Rajendran A. Acellular dental pulp and allogeneic cells for strategic pulp regeneration. Tissue Cell. (2026) 100:103369. 10.1016/j.tice.2026.103369 41687189

[94] Mayya A Chatra A Dsouza V Seetharam RN Acharya SR Vasanthan KS. Biomaterials to biofabrication: advanced scaffold technologies for regenerative endodontics. Biomed Phys Eng Express. (2026) 12(1):012001. 10.1088/2057-1976/ae2b75 41380175

[95] Dissanayaka WL Zhang C. Scaffold-based and scaffold-free strategies in dental pulp regeneration. J Endod. (2020) 46(9S):S81–9. 10.1016/j.joen.2020.06.022 32950199

[96] Raddall G Mello I Leung BM. Biomaterials and scaffold design strategies for regenerative endodontic therapy. Front Bioeng Biotechnol. (2019) 7:317. 10.3389/fbioe.2019.00317 31803727 PMC6874017

[97] Wang Y Mao J Wang Y Wang R Jiang N Hu X. Odontogenic exosomes simulating the developmental microenvironment promote complete regeneration of pulp-dentin complex *in vivo*. J Adv Res. (2025) 76:405–21. 10.1016/j.jare.2024.12.048 39765328 PMC12793753

[98] Liu H Zhang H Yang W Chen H Wang J Chang F. Natural biomaterials for osteochondral repair: from source to strategy. Adv Healthc Mater. (2026) 15(27):e71344. 10.1002/adhm.71344 42299644 PMC13378495

[99] Vatankhah M Najary S Dianat O. Clinical, radiographic, and histologic outcomes of regenerative endodontic treatment in human immature teeth using different biological scaffolds: a systematic review and meta-analysis. Curr Stem Cell Res Ther. (2024) 19(4):611–27. 10.2174/1574888X17666220903141155 36056831

[100] Asgary S Eghbal MJ Shamszadeh S. Advancements and challenges in stem/progenitor cell transplantation for dentin-pulp regeneration: a systematic review of animal studies (part I). Am J Stem Cells. (2024) 13(3):110–31. 10.62347/HENE2422 39021372 PMC11249669

[101] Yamada Y Nakamura-Yamada S Konoki R Baba S. Promising advances in clinical trials of dental tissue-derived cell-based regenerative medicine. Stem Cell Res Ther. (2020) 11(1):175. 10.1186/s13287-020-01683-x 32398041 PMC7218566

